# A Preliminary Study of the Algicidal Mechanism of Bioactive Metabolites of *Brevibacillus laterosporus* on *Oscillatoria* in Prawn Ponds

**DOI:** 10.1155/2014/869149

**Published:** 2014-03-09

**Authors:** Wen Jia, Xianghu Huang, Changling Li

**Affiliations:** Department of Aquaculture, Fishery College, Guangdong Ocean University, East Huguangyan, Mazhang District, Zhanjiang City, Guangdong Province 524088, China

## Abstract

The algae, *Oscillatoria*, is commonly found in prawn ponds and can lead to reduced productivity. We examined metabolites of the bacteria *Brevibacillus laterosporus* for algicidal qualities. To determine the possible algicidal mechanisms of these bioactive metabolites, different amounts of sterile filtrate of bacterial suspensions were added to cultures containing *Oscillatoria*. The dry weight, the concentrations of chlorophyll-a (chl-a), phycobiliprotein (PC, phycocyanin; APC, allophycocyanin; PE, phycoerythrin), and MDA (malondialdehyde) and the activities of SOD (superoxide dismutase), POD (peroxidase), and CAT (catalase) of algae were measured during the algicidal application. The results showed that lower concentrations of the sterile filtrate (addition ≤ 4 mL) accelerated the growth rate of *Oscillatoria*, but significant inhibition and lysis were observed with higher concentrations (addition ≥ 8 mL). In two trials (the additions were 8 mL and 10 mL, respectively), the algal dry weights were reduced by 26.02% and 45.30%, and the chl-a concentrations were decreased by 46.88% and 63.73%, respectively, after seven days. During the algicidal treatment, the concentrations of PC, APC, PE, and MDA and the activities of SOD, POD, and CAT were significantly increased in the early cultivation and declined quickly at later stages. Finally, the algae-lysing mechanism of the bioactive metabolites of the bacteria *Brevibacillus laterosporus* on *Oscillatoria* had been proposed.

## 1. Introduction

As the water quality of prawn ponds is steadily degrading, exploring the environmental and economic impacts caused by microalgae, especially by* Oscillatoria*, has been of increasing interest. To date, physical means (such as microfiltration membrane, optical isolation, and air flotation processes to remove algae) and chemical agents (algicides, flocculant precipitation, and mineral flocculation) are commonly used in controlling and removing the harmful algae [1–3]. However, these methods are not ideal for four reasons [4–8]. First, they are time-consuming. Second, they are costly. Third, the effects are small. Finally and most importantly, they may cause secondary pollution. Algae-lysing bacteria [9–12], as an important component in aquatic ecosystems, can play an important role in improving the water quality in culture ponds. So far, several studies on algae-lysing bacteria have been reported in the literature, including four aspects of isolation and identification of algae-lysing bacteria and descriptions of the algicidal phenomenon [13–16], exploration of the mode of bacterial lysing [17–20], and isolation and identification of the algae-lysing active substances [12, 21, 22]. However, the study of the algicidal mechanism is still limited.

The algae-lysing bacteria used in this experiment were extracted from a sea mud sample collected in the intertidal zone of Zhanjiang coastal park. According to 16S rDNA sequence analysis, it was found to be* Brevibacillus laterosporus*. We studied the algicidal effect of the bioactive metabolites of the bacteria on* Oscillatoria,* which is common in prawn ponds. The objectives of this paper were to (i) determine the concentration at which the bioactive metabolites demonstrate the best algicidal effect, (ii) determine the effects of the bioactive metabolites on* Oscillatoria*, and (iii) explore the algicidal mechanism.

## 2. Materials and Methods

### 2.1. The Species of Algae Used in the Study

*Oscillatoria* provided by the laboratory of ecology of water area and aquaculture environment, Guangdong Ocean University (Zhanjiang, China), was extracted from the higher-elevation pond belonging to Zhanjiang Zhonglian culturing Co., Ltd.

### 2.2. The Species of Bacteria Used in the Study

The bacteria used in the study were isolated from a sea mud sample collected 5 to 10 cm beneath the surface of the intertidal zone in Zhanjiang coastal park. It was identified as* Brevibacillus laterosporus* by physiological and biochemical indexes and an analysis of 16S rDNA. This bacterial strain was preserved by the laboratory of ecology of water area and aquaculture environment, Guangdong Ocean University.

### 2.3. Culture Medium

The algal medium used was BG11 culture solution [23], while the bacterial culture medium used was nutrient broth (NB) [24].

### 2.4. The Preculturing of Algae

A microalgae suspension with 3-percent inoculum was added to fresh sterile BG11 medium. The cultural conditions were a temperature of 28°C, an illumination intensity of 54 *μ*mol·m^−2^s^−1^, a salinity of 28 ppt, a light-dark cycle of 14 h : 10 h, and a pH of 7.8 ± 0.2. In the seven days preculture, the bottle of the suspension was shaken three times a day at regular intervals, maintaining an optical density-OD_660_ between 0.22 and 0.26. The inoculation was conducted using sterile technique.

### 2.5. Germiculture and Extraction of Sterile Filtrate

We extracted two loops of the algicidal bacteria, cultured on an agar slant, and inoculated fresh NB medium. The initial bacterial suspension was cultured for 12 hours at a rate of 120 r·min^−1^ at 30°C. Then the suspension was added to new fresh NB medium at a final dilution of three percent. After another 30 hours of shaking, we obtained a bacterial fermentative solution. Next, we centrifuged the fermentative solution at the rate of 10,000 r·min^−1^, 4°C for 20 min. The supernatant was twice filtered through a membrane filter (0.22 *μ*m). We determined the presence of any bacteria by plating. When there were no bacteria in the filtrate, the optical density-OD_600_ of the sterile filtrate was measured and determined to be 0.039.

### 2.6. The Design of Experiment

The sterile filtrate was added at 6 different levels, 0, 2, 4, 6, 8, and 10 mL, to an* Oscillatoria* suspension that had been precultured for seven days. Each solution was then diluted to 100 mL with fresh BG11 medium. Each treatment was conducted in triplicate.

### 2.7. The Impact on the Dry Weight of Algal Cells

Algal cells were collected at the beginning of the experiment and on the seventh day by filtering 80 mL solution of each group using medium-speed qualitative filter papers that had been dried for 24 hours at 60°C. The filter papers with the algal cells were also dried for 24 hours at 60°C. The dry weight of the algal cells was calculated using (1):

(1) $$\begin{array}{c} M=M_{T}-M_{0}, \end{array}$$

where *M* represents the value of dry weight of the algal cells and *M*_0_ and *M*_*T*_ are the dry weights of the filter paper in grams and of the filter paper with the algal cells, respectively.

### 2.8. The Impact on the Concentration of Chlorophyll-a (chl-a) of Algal Cells

An aliquot of 10 mL of the dilution was removed daily and then centrifuged at a rate of 4,000 r·min^−1^ for 10 min and then the supernatant was decanted. An additional 5 min of centrifugation was performed to extract the dry algal cells. An identical operation was conducted on the supernatant. The algal cells were ruptured using a two-day cryogenic treatment in a −20°C freezer. Next, 10 mL of 90% acetone was added. The acetone solution was well-mixed to extract the chl-a and placed in a 4°C refrigerator in total dark. After 24 hours, the solution underwent a third centrifugation for 10 min at a rate of 4,000 r·min^−1^. Then the absorbance of the supernatant was determined at the wavelengths of 630, 647, 664, and 750 nm, using acetone as a reference. The mass concentration of chl-a (mg·L^−1^) was determined using the following [25]:

(2) ρchl-a=[11.85(D664−D750)−1.54(D647−D750)     −0.08(D630−D750)]×V1V2·d,

where *D*_664_, *D*_647_, *D*_630_, and *D*_750_ are the values of absorbance of the supernatant at the wavelengths of 630, 647, 664, and 750 nm, respectively. *V*_1_ is the volume after adding 90% acetone (mL), *V*_2_ is the volume removed from the dilution (L), and *d* is optical path length of the cuvette (cm).

### 2.9. The Impact on the Content of Phycobiliprotein of Algae Cells

An aliquot of 10 mL of the sample was extracted daily and then centrifuged at the rate of 4,500 r·min^−1^ for 20 min, and then the supernatant was decanted. The aliquot was well-mixed by adding 10 mL pH 7.0 phosphate buffer (0.1 mol·L^−1^) and then placed in a −20°C refrigerator for one hour and then thawed at 4°C. The mixture was centrifuged at a rate of 4,500 r·min^−1^ for 10 min, and then absorbance of the supernatant was determined at the wavelengths of 652, 620, and 562 nm, using phosphate buffer as a reference. The concentrations of phycocyanin (PC), allophycocyanin (APC), and phycoerythrin (PE) were calculated according to (3) to (5), respectively [26]:

(3) $$\begin{array}{c} C_{\text{PC}}=0.187A_{615}-0.089A_{652} \end{array}$$

(4) $$\begin{array}{c} C_{\text{APC}}=0.196A_{652}-0.041A_{615} \end{array}$$

(5) $$\begin{array}{c} C_{\text{PE}}=0.104A_{562}-0.253C_{\text{PC}}-0.088C_{\text{APC}}, \end{array}$$

where *A*_652_, *A*_620_, and *A*_562_ are the values of absorbance of the crude supernatant on the wavelengths of 652, 620, and 562 nm, respectively. *C*_PC_, *C*_APC_, and *C*_PE_ are the concentrations in mg·L^−1^ of PC, APC, and PE, respectively.

### 2.10. The Impact on Malondialdehyde (MDA) and the Activities of Superoxide Dismutase (SOD), Peroxidase (POD), and Catalase (CAT)

An aliquot was extracted each day and then centrifuged at the rate of 4,500 r/min for 20 min, then the supernatant was decanted. To obtain algal cells, we added 0.9 mL phosphate buffer (pH 7.4, 0.1 mol·L^−1^) to 0.1 g of algal cells and then ground them in an ice-bath for five min (10-percent tissue homogenate). The tissue homogenate was then centrifuged at the rate of 10,000 r·min^−1^ and 4°C for 10 min. The supernatant was used for the determination of enzyme activity. The kits used in this procedure were from Nanjing Jiancheng Bioengineering Institute. Measurements of enzyme activity were conducted with the aid of a Bioteku Quart All Band Microplate Readers.

### 2.11. Data Analysis

Statistical tests were conducted using SPSS (version 17). One-way ANOVA was used to examine the algicidal effects of the sterile filtrate on* Oscillatoria*. If the ANOVA showed significant differences, a multiple comparison (LSD) test was used to examine differences between groups.

## 3. Results

### 3.1. The Impact on the Dry Weight of Algal Cells

**Figure 1:**
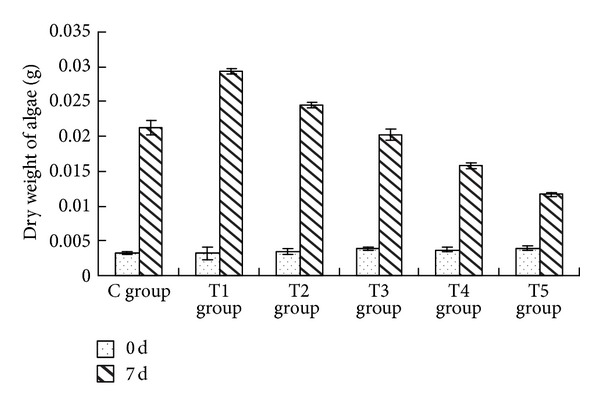
Effects of the sterile filtrate of algae-lysing bacteria in different concentrations on the dry weight of* Oscillatoria* (C group is the control group, the T1 group with 2 mL sterile filtrate, the T2 group with 4 mL sterile filtrate, the T3 group with 6 mL sterile filtrate, the T4 group with 8 mL sterile filtrate, and the T5 group with 10 mL sterile filtrate).

The sterile filtrate of algicidal bacteria at different concentrations showed a statistically significant impact on the dry weight of* Oscillatoria* (ANOVA result, *F* = 311.312, *P* < 0.01) (Figure 1).

Compared with the control group, the dry weight of* Oscillatoria* in the Trial 1 (T1) group (the addition of the sterile filtrate was 2 mL) and Trial 2 (T2) group (the addition of the sterile filtrate was 4 mL) increased by 38.24% and 15.52%, respectively. The lower concentrations of the sterile filtrate, the greater increase in the dry weight of* Oscillatoria*. However, in the Trial 4 (T4) group (the addition of the sterile filtrate was 8 mL) and the Trial 5 (T5) group (the addition of the sterile filtrate was 10 mL), the dry weight decreased by 26.02% and 45.30%, respectively, compared to the control group. There was an increasing reduction in the dry weight of* Oscillatoria* with the greater concentrations of the sterile filtrate. The dry weight of the Trial 3 (T3) group (the addition of the sterile filtrate was 6 mL) was approximately equal to the control group.

Multiple comparison (LSD) tests showed that except for the T3 group, the differences between other test groups and control group were all statistically significant. Additionally there were highly significant differences among the test groups for dry weight.

### 3.2. The Impact on the Concentration of chl-a of Algal Cells

**Figure 2:**
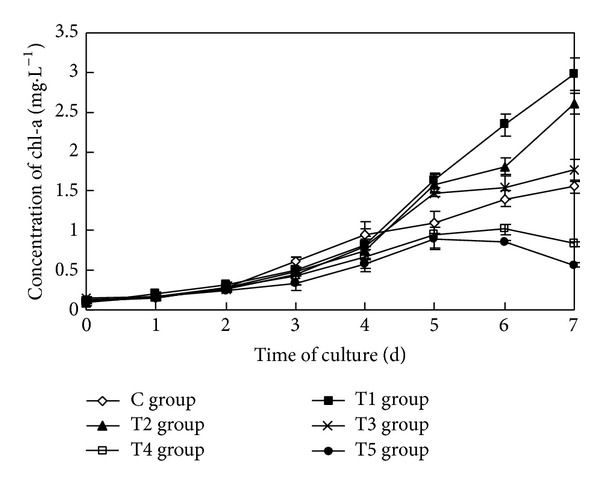
Effects of the sterile filtrate of algae-lysing bacteria in different concentrations on the concentration of chl-a of* Oscillatoria* (C group is the control group, the T1 group with 2 mL sterile filtrate, the T2 group with 4 mL sterile filtrate, the T3 group with 6 mL sterile filtrate, the T4 group with 8 mL sterile filtrate, and the T5 group with 10 mL sterile filtrate).

The sterile filtrate of the algicidal bacteria at different concentrations had a statistically significant impact on the concentration of chl-a of* Oscillatoria* (ANOVA result, *F* = 188.061, *P* < 0.01) (Figure 2).

The concentration of chl-a of* Oscillatoria* in the control group increased steadily during the course of the study, up to 1.561 mg/L seven days later. In the T1 group (2 mL) and the T2 group (4 mL), the concentration of chl-a of* Oscillatoria* increased four days later, achieving 2.981 mg·L^−1^ and 2.610 mg·L^−1^, respectively, seven days later and increasing by 90.94% and 67.15% compared to the control group, respectively. The concentration of chl-a of* Oscillatoria* in the T3 group (6 mL) increased slightly relative to the control group and increased by only 12.75%. However, the concentration of chl-a in the T4 group (8 mL) and the T5 group (10 mL) was consistently lower than that of the control group and declined five days later, to 0.829 mg·L^−1^ and 0.566 mg·L^−1^, a decrease of 46.88% and 63.73% relative to the control group, respectively.

Multiple comparison (LSD) tests showed that except for the T3 group, the differences between the other test groups and the control group were all statistically significant. There was no significant difference between the T4 group and the T5 group; however, statistically significant differences existed among the other test groups.

### 3.3. The Impact on the Concentration of Phycobiliproteins of Algal Cells

**Figure 3:**
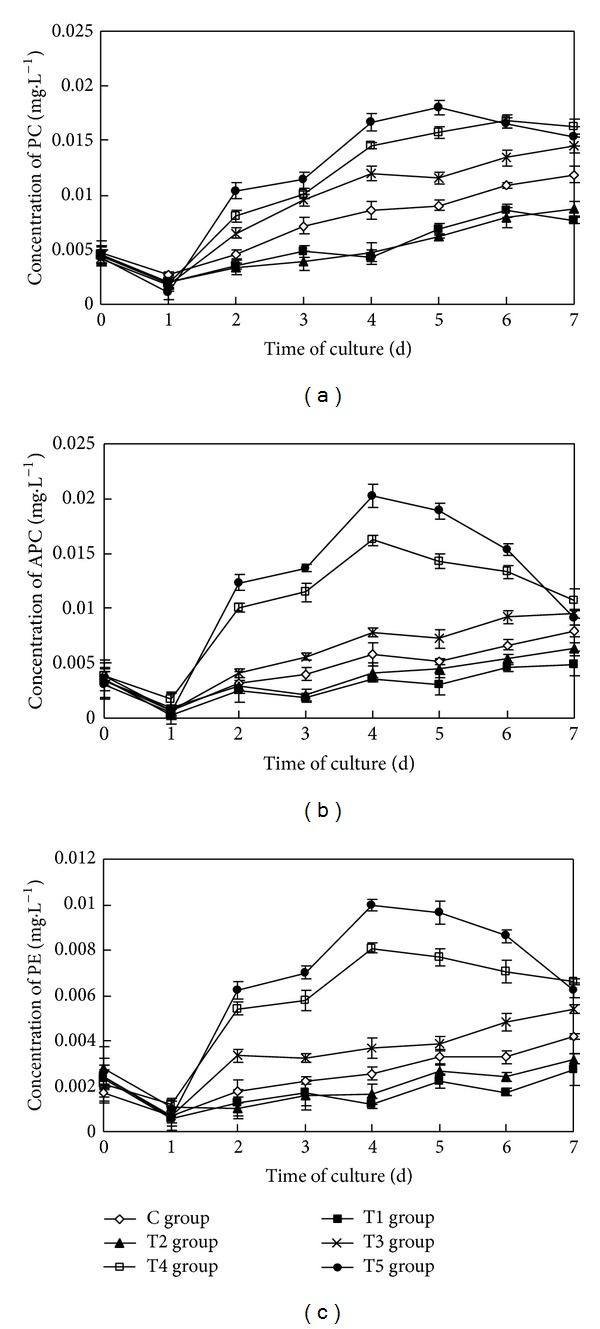
Effects of the sterile filtrate of algae-lysing bacteria in different concentrations on the phycobiliprotein concentrations of* Oscillatoria *for (a) the concentration of PC (phycocyanin), (b) the concentration of APC (allophycocyanin), and (c) the concentration of PE (phycoerythrin) (C group is the control group, the T1 group with 2 mL sterile filtrate, the T2 group with 4 mL sterile filtrate, the T3 group with 6 mL sterile filtrate, the T4 group with 8 mL sterile filtrate, and the T5 group with 10 mL sterile filtrate).

The sterile filtrate of the algicidal bacteria at different concentrations had a statistically significant impact on the concentration of phycobiliproteins (PC, APC, and PE) of* Oscillatoria* (ANOVA result, *F*_PC_ = 277.658, *P*_PC_ < 0.01; *F*_APC_ = 259.484, *P*_APC_ < 0.01; *F*_PE_ = 395.869, *P*_PE_ < 0.01, resp.) (Figure 3).

During the course of the study, the concentrations of three phycobiliproteins of* Oscillatoria* in the control group increased steadily, up to 0.012 mg·L^−1^, 0.008 mg·L^−1^, and 0.004 mg·L^−1^ seven days later, respectively. In the T1 group and the T2 group, the concentrations of the three phycobiliproteins (PC, APC, and PE) increased slowly but were consistently lower than in the control group. Seven days later, the contents of PC, APC, and PE in the T1 group and the T2 group accounted for 64.61%, 62.38%, 65.06% and 73.58%, 79.20%, 76.29% of the concentrations of the control group, respectively. The concentrations of PC, APC, and PE in the T3 group increased slightly relative to the control group, increasing by 22.66%, 20.41%, and 28.88%, respectively. The concentrations of PC, APC, and PE in the T4 group and the T5 group all increased initially and then declined. The concentrations of PC, APC, and PE in the T4 group and the T5 group remained 37.33%, 35.58%, 57.74% and 29.33%, 15.26%, 48.90% higher than that of the control group seven days later, respectively. The concentrations of PC, APC, and PE in the T4 group and the T5 group increased by 266.16%, 180.10%, 216.98% and 260.76%, 198.73%, 158.35% from the initial values, respectively.

Multiple comparison (LSD) tests showed that the differences between the test groups and the control group were all statistically significant for the three phycobiliproteins. Meanwhile, there was no significant difference between the T1 group and the T2 group, but statistically significant differences existed among the other test groups for the three phycobiliproteins.

### 3.4. The Impact on the Concentration of MDA of Algae Cells

**Figure 4:**
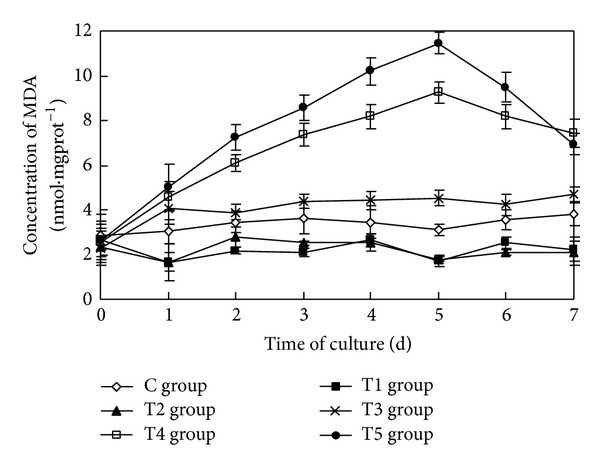
Effects of the sterile filtrate of algae-lysing bacteria in different concentrations on MDA (malondialdehyde) concentrations of* Oscillatoria* (C group is the control group, the T1 group with 2 mL sterile filtrate, the T2 group with 4 mL sterile filtrate, the T3 group with 6 mL sterile filtrate, the T4 group with 8 mL sterile filtrate, and the T5 group with 10 mL sterile filtrate).

The sterile filtrate of the algicidal bacteria at different concentrations had a statistically significant impact on the concentration of MDA of* Oscillatoria* (ANOVA result, *F* = 415.093, *P* < 0.01) (Figure 4).

During the course of the study, the concentration of MDA of* Oscillatoria* in the control group was always maintained at a low level. Although there were small fluctuations in the concentration of MDA of* Oscillatoria* in the T1 group and the T2 group, the concentrations of MDA were lower than in the control group. In the T3 group, the concentration of MDA increased for one day and then remained at a higher level than that of the control group and also fluctuated slightly. The concentration of MDA in the T4 group and the T5 group increased initially, with a greater concentration caused by a higher rate of increase. The concentration of MDA in the T4 group and the T5 group reached their maximums, 9.25 nmol·mgprot^−1^ and 11.45 nmol·mgprot^−1^, respectively, five days after the initiation of the study. The values increased by 197.67% and 268.72% relative to the control group, respectively. From then on, the concentrations of MDA in the T4 group and the T5 group began to decline; the rate of decrease was greater as the concentration of sterile filtrate increased. Seven days later, the concentrations of MDA in the T4 group and the T5 group were still higher than in the control group.

Multiple comparison (LSD) tests showed that the results were the same as that for the three phycobiliproteins.

### 3.5. The Impact on the Activity of SOD of Algal Cells

**Figure 5:**
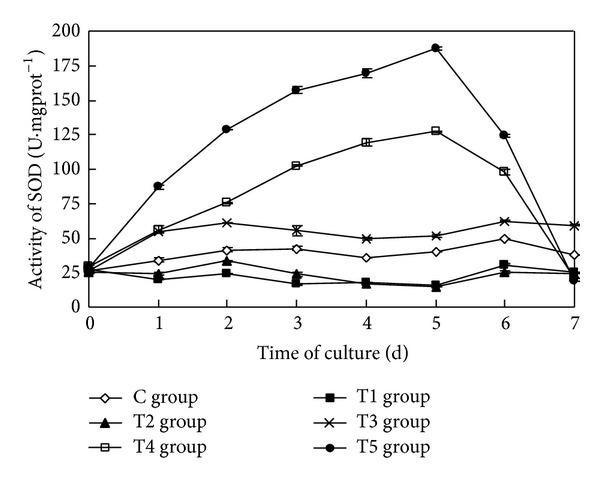
Effects of the sterile filtrate of the algae-lysing bacteria in different concentrations on the SOD (superoxide dismutase) activity of* Oscillatoria* (C group is the control group, the T1 group with 2 mL sterile filtrate, the T2 group with 4 mL sterile filtrate, the T3 group with 6 mL sterile filtrate, the T4 group with 8 mL sterile filtrate, and the T5 group with 10 mL sterile filtrate).

The sterile filtrate of the algicidal bacteria at different concentrations had a statistically significant impact on the activity of SOD of* Oscillatoria* (ANOVA result, *F* = 26357.020, *P* < 0.01) (Figure 5).

During the course of the study, the activity of SOD of* Oscillatoria* in the control group always remained at a low level. Although there were small fluctuations in the activities of SOD of* Oscillatoria* in the T1 group and the T2 group, the activities of SOD were lower than that in the control group. In the T3 group, the activity of SOD increased in one day and then remained higher than that of the control group, but fluctuated slightly. The activities of SOD in the T4 group and the T5 group increased initially, with the greater concentration caused by a higher rate of increase. The activities of SOD in the T4 group and the T5 group reached their maximums, 126.92 U·mgprot^−1^ and 187.51 U·mgprot^−1^, respectively, five days after the initiation of the study. Their values increased by 218.94% and 371.21% relative to the control group, respectively. From then on, the activities of SOD in the T4 group and the T5 group began to decline, and the rate of decrease was greater as the concentration of sterile filtrate increased. Seven days later, the activities of SOD in the T4 group and the T5 group decreased by 45.37% and 49.47% than in the control group, respectively.

Multiple comparison (LSD) tests showed that the differences between the test groups and the control group were all statistically significant. At the same time, there were statistically significant difference between the T1 group and the T2 group, and additional statistically significant differences existed among the other test groups.

### 3.6. The Impact on the Activity of POD of Algae Cells

**Figure 6:**
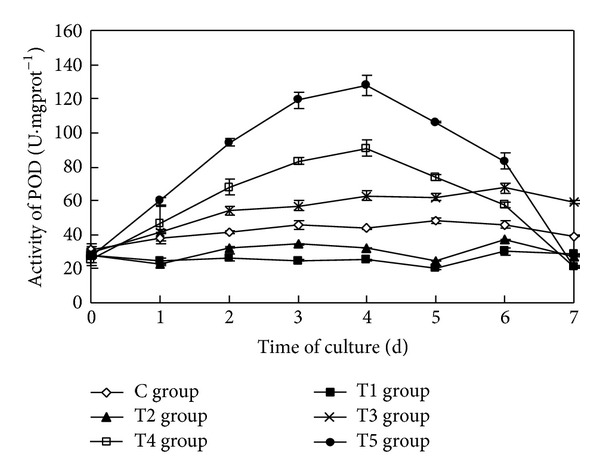
Effects of the sterile filtrate of the algae-lysing bacteria in different concentrations on the POD (peroxidase) activity of* Oscillatoria* (C group is the control group, the T1 group with 2 mL sterile filtrate, the T2 group with 4 mL sterile filtrate, the T3 group with 6 mL sterile filtrate, the T4 group with 8 mL sterile filtrate, and the T5 group with 10 mL sterile filtrate).

The sterile filtrate of the algicidal bacteria at different concentrations had a statistically significant impact on the activity of POD of* Oscillatoria* (ANOVA result, *F* = 423.504, *P* < 0.01) (Figure 6).

During the course of the study, the activity of POD of* Oscillatoria* in the control group remained at a low level, but in the T1 group and the T2 group, the activities of POD were distinctly lower than that in the control group. In the T3 group, the activity of POD increased in the first two days and then remained higher than the control group, but fluctuated slightly. The activities of POD in the T4 group and the T5 group increased initially, with a greater concentration caused by a higher rate of increase. The activities of POD in the T4 group and the T5 group reached their maximums, 91.00 U·mgprot^−1^ and 127.91 U·mgprot^−1^, respectively, four days after the commencement of the experiment. Their values increased by 107.65% and 191.89% relative to the control group, respectively. From then on, the activities of POD in the T4 group and the T5 group declined substantially; the rate of decrease was greater as the concentration of the sterile filtrate increased. Seven days later, the activities of POD in the T4 group and the T5 group decreased by 46.23% and 46.41% than in the control group, respectively.

Multiple comparison (LSD) tests showed that the results were the same as that of the SOD.

### 3.7. The Impact on Activity of CAT of Algae Cells

**Figure 7:**
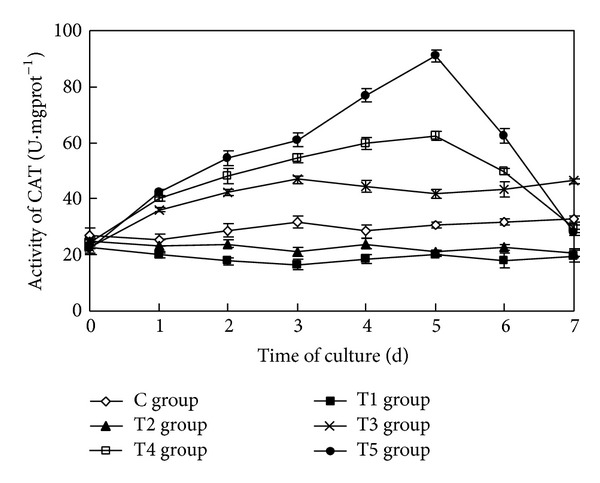
Effects of the sterile filtrate of the algae-lysing bacteria in different concentrations on the CAT (catalase) activity of* Oscillatoria* (C group is the control group, the T1 group with 2 mL sterile filtrate, the T2 group with 4 mL sterile filtrate, the T3 group with 6 mL sterile filtrate, the T4 group with 8 mL sterile filtrate, and the T5 group with 10 mL sterile filtrate).

The sterile filtrate of algicidal bacteria at different concentrations had an extremely significant impact on the activity of CAT of* Oscillatoria* (ANOVA result, *F* = 1146.197, *P* < 0.01) (Figure 7).

During the course of the study, the activity of CAT of* Oscillatoria* in the control group always remained at a low level. In the T1 group and the T2 group, the activities of CAT were distinctly lower than that in the control group. In the T3 group, the activity of CAT increased initially and then remained higher than the control group, but fluctuated slightly. The activities of CAT in the T4 group and the T5 group increased initially, with the greater concentration caused by a higher rate of increase. The activities of CAT in the T4 group and the T5 group reached their maximums, 62.42 U·mgprot^−1^ and 91.10 U·mgprot^−1^, respectively, five days after the initiation of the study. Their values increased by 102.54% and 195.64% relative to the control group, respectively. From then on, the activities of CAT in the T4 group and the T5 group declined sharply; the rate of decrease was greater as the concentration of sterile filtrate increased. Seven days later, the activities of CAT in the T4 group and the T5 group decreased by 10.31% and 14.20% than in the control group, respectively.

Multiple comparison (LSD) tests showed that the results were the same as that of the three phycobiliproteins and the MDA.

## 4. Discussion

### 4.1. The Impact of Different Concentrations of the Sterile Filtrate on the Photosynthesis of* Oscillatoria*

In the photosynthesis system of* Oscillatoria*, chl-a is the central pigment, while PC, APC, and PE play important roles in capturing light and transferring the light energy. The impact of the algicidal bacteria or its bioactive metabolites on photosynthesis is measurable, which could induce changes in the photosynthesis system, specifically electron transport as well as photosynthetic efficiency. It was discovered that the bioactive metabolites of* Brevibacillus laterosporus*, B1 17, had a statistically significant algicidal effect on cyanobacteria (*Anabaena* sp.,* Nostoc, Microcystis*) that could also interfere with the normal operation of the photosynthetic component in the electron transport chain receptor binding domain, belonging to photosynthesis II; in addition, the bioactive metabolites degraded phycobilin and altered the organization structure of algal cells [27]. The bioactive metabolites of L8 bacterium distinctly enhanced the contents of carotenoid, phycocyanin, and allophycocyanin of* Anabaena flos-aquae*. Concurrently, the content of chl-a, photochemical efficiency in the photosystem, and the output of photosynthetic electrons showed an obvious decline [28]. And another researcher also came to the conclusion that the damage in the photosynthesis system of the algal cells was caused by the bacterium and had given rise to the algicidal effect [29].

The results showed that the impacts of different concentrations of the sterile filtrate on the photosynthesis of* Oscillatoria* varied. Lower concentrations of sterile filtrate (an addition of no more than 4 mL) increased the content of chl-a, while the contents of PC, APC, and PE decreased simultaneously. Higher concentrations (an addition of no less than 8 mL) decreased the content of chl-a and the contents of PC, APC, and PE increased at first and decreased later. However, the values of PC, APC, and PE were consistently higher than normal. The underlying reason was that when the sterile filtrate was at lower concentrations, the sterile filtrate concentrations were able to reduce the relative proportion of light-harvesting proteins existing in algal cells. Meanwhile, the capture and the utilization of energy turned out to be more efficient in that it avoided excessive damage, and in turn, photosynthesis was enhanced [30–32]. However, higher concentrations damaged the D1 protein in photosystem II (PS II) within the* Oscillatoria* cells and then blocked the normal transfer of light energy among PE to PC to APC to Chl-a, severely affected the distribution of light energy, altered the contents of each pigment and their relative proportions, and inhibited photosynthesis [33]. This dose effect that high concentrations cause inhibition while low concentrations lead to promotion, similar to the effect of growth hormone, has been reported in several studies [34, 35]. In addition, studies showed that photosynthesis could be enhanced by reducing the proportion of light-harvesting pigment [32, 36]. According to the results of this study, we maintain that photosynthesis could be enhanced by reducing the proportions of light-harvesting pigments; conversely, photosynthesis could be inhibit by increasing the proportions of light-harvesting pigments.

### 4.2. The Impact of Different Concentrations of Sterile Filtrate on the Concentration of MDA of Algal Cells

MDA is the final product of lipid peroxidation, and its concentration can reflect on the degree of cell membrane lipid peroxidation and the stress tolerance of plants [37]. The three kinds of algicidal bacteria, DC10, DC22, and DC-P can induce membrane lipid peroxidation in* Aphanizomenon flos-aquae*, FACHB 943, maintaining a high level of MDA [38]. In addition, research has shown that the extracellular active substances of algicidal bacteria S7 (*Chryseobacterium*) can induce membrane lipid peroxidation in* Microcystis aeruginosa* 905, resulting in an increase in the concentration of MDA in algal cells at an early stage, followed by a decline five days later [39].

In this study, the impacts of different concentrations of sterile filtrate on the concentration of MDA in* Oscillatoria* cells were different. The concentration of MDA in* Oscillatoria* cells remained at an extremely low level when the concentrations of sterile filtrate were low (the addition being no more than 4 mL). When the concentrations of sterile filtrate were increased (the addition being no less than 6 mL), the concentration of MDA in the algal cells increased at first then declined. This implies that low concentrations of the sterile filtrate of this bacterium reduced the damage to* Oscillatoria*, strengthened the cell membrane structure, and lowered the membrane lipid peroxidation. Alternately, when the concentration of the sterile filtrate is at high levels, the membrane lipid peroxidation significantly increased as did the concentration of MDA. That the concentration of MDA declined at a later stage may be related to severe damage of the membrane system of the algal cells and the occurrence of cytoclasis [39].

### 4.3. The Impact of Different Concentrations of Sterile Filtrate on the Antioxidant Enzyme System of Algal Cells

Plants are subjected to various forms of oxidative stress in nature. In the process of adaptation, they have developed an antioxidation system, including a series of antioxidant enzymes and other antioxidant substances, to eliminate excess reactive oxygen species, strengthen oxidation resistance, protect the redox status of cells, maintain the reactive oxygen within the cells at a low level, tolerate the damage caused by an adverse environment, and ensure normal growth and metabolism [38]. SOD plays a major role in the process of eliminating reactive oxygen species and is capable of converting superoxide anions (O_2_^−^) into H_2_O_2_ and divalent oxygen rapidly [40]. Then, with the help of CAT and POD, H_2_O_2_ transforms into water and divalent oxygen, which prevents the damage caused by the accumulation of H_2_O_2_ in the body [41, 42]. Tang et al. [43] reported that the algae-lysing substances of actinomycetes L74 enabled the production of SOD, POD, and CAT in the antioxidant enzyme system of* Microcystis aeruginosa* FACHB 905 to rise substantially at early stages, but it decreased considerably at later stages. Fu et al. [44] found that the activities of SOD and CAT in the antioxidant enzyme system of* Alexandrium tamarense* ATGD98-006 increased as the concentration of the supernatant of the algicidal bacteria BS03 (*Microbulbifer* sp.) increased; however, as the time of the treatment was extended, activity increased at first and then declined.

The impacts of different concentrations of sterile filtrate on the antioxidant enzyme system in* Oscillatoria* cells shown in this study were different. Low concentrations of sterile filtrate (the addition was no more than 4 mL) maintained the activity of the antioxidant enzymes, such as SOD, POD, and CAT, consistently lower than that in the control group. As the concentration of sterile filtrate increased (the addition was no less than 6 mL), the activity of SOD, POD, and CAT in* Oscillatoria* cells was enhanced and increased at first then declined. We suggest that low concentrations of sterile filtrate worked as protective agents of the* Oscillatoria* cells, reduced the amount of reactive oxygen species produced by normal metabolism, maintained reactive oxygen within the cells at a low level, and led to an activity of antioxidant enzymes that were all lower than normal. As the concentration of sterile filtrate increased, a slight stress occurred, resulting in more reactive oxygen species in algal cells, and the antioxidant enzyme system was initiated. At the same time, the activities of SOD, POD, and CAT increased, and the ability to remove reactive oxygen was reinforced. Thus, the metabolism of reactive oxygen was maintained and the stress reduced. However, the stress caused by high concentrations of sterile filtrate was greater, and over time, the algal cells were subjected to excessive oxidative damage and the reactive oxygen species were beyond their reducing capacity. The activity of the antioxidant enzymes became depressed; thus the antioxidant system was damaged, and the activity of the antioxidant enzymes declined rapidly [38, 43, 45].

### 4.4. The Algicidal Mechanism of the Bioactive Metabolites of the Bacteria on* Oscillatoria*

The method with which algicidal bacteria acts on algae cells and the possible algicidal mechanism could be divided into five classes: direct contact [46], releasing a substance that kills the algae [47], competing for nutrients [48], formation of a bacteria adhesive film, and entering into the algae cells [49]. The results of this study suggest that the* Brevibacillus laterosporus* produced an algicidal effect on* Oscillatoria* by means of an extracellular secretion, specifically when the extracellular secretion accumulated to a certain concentration. Evidently, this mechanism was indirect.

With respect to this indirect means, it is suggested that a substance acted on the process of metabolism in the algae, for example, depressing cell wall synthesis, blocking a respiratory chain, depressing sporulation, and so on, to depress cell processes or kill the algae cells [50]. According to the measurements of the growth and physiological indexes of* Oscillatoria* in this experiment, the algicidal mechanism is proposed as follows. Firstly, algicidal substances acted on the algae cells, triggering membrane lipid peroxidation of the algal cells, elevating the level of biofilm esterification, and destroying the membrane system by increasing the selective permeability of the cell membrane. Second, the relative proportion of each pigment was changed, the transmission and distribution of light energy were impacted, and the photosynthesis system was damaged. Meanwhile, the antioxidant enzyme system was damaged and the activities of various antioxidases were inhibited. Finally, the algicidal substances led to the loss of normal physiological and metabolic function and thus the cells of* Oscillatoria* lysed and died and as a result, the biomass declined [28, 39].

### 4.5. The Biocontrol of Cyanobacteria and Prawn Healthy Breeding

The eutrophication of prawn ponds is becoming increasingly severe, which impacts the community composition of the phytoplankton in the culture ponds.* Oscillatoria*, owing to its extremely strong vitality and competitiveness, has become the dominant species in prawn culture ponds. It impacts the prawn in two ways. First, it consumes a large amount of the dissolved oxygen that is essential for the growth of the prawns. Second, the nervous system of the prawn is damaged by the by-products of* Oscillatoria* [51], such as microcystins (MC) and analogues [52] and proteins [53] as well as other metabolites. Considering the side effects of the physical and chemical methods, He et al. [54] discussed the impact of environmental factors on the growth of* Oscillatoria* and their objective was to investigate the depression of growth of* Oscillatoria* by the regulation of environmental factors, but this was still not so effective. With the development of the algal biological control technology, recent studies have indicated that adding beneficial bacteria, algae, or the composition of bacteria and algae in prawn ponds, the cycling of substances and energy could be accelerated [55, 56], the growth of pathogenic microorganism could be depressed, and the survival rate of the prawn could be increased [55, 57–59], also increased was the activity of immunoperoxidase and disease resistance of prawns [60]. In this study, we found that when the additions of sterile filtrate were no less than 8 mL (the initial concentrations were no less than 8%), the cells of* Oscillatoria* were ruptured and readily decomposed. Thus, we presented an efficient biological technique to control* Oscillatoria* in prawn ponds.

## 5. Conclusion

In this study, effects of different amounts of sterile filtrate of bacterial suspensions (*Brevibacillus laterosporus*) added to cultures containing* Oscillatoria* in prawn ponds were researched. Lower concentrations of the sterile filtrate (addition ≤ 4 mL) accelerated the growth rate of* Oscillatoria*, but significant inhibition and lysis were observed with higher concentrations (addition ≥ 8 mL). In two trials with higher concentration of the sterile filtrate, the algal dry weights and the chl-a concentrations were lower than the control group during the algicidal treatment; both the concentrations of PC, APC, PE, and MDA and the activities of SOD, POD, and CAT were significantly increased in the early cultivation and declined quickly at later stages. So the algicidal mechanism was proposed: acting on the cells of* Oscillatoria*, bioactive metabolites of* Brevibacillus laterosporus* destroyed the membrane system, the photosynthesis system, and the antioxidant enzyme system gradually. Finally the cells of* Oscillatoria* were lysed resulting from the loss of normal physiological and metabolic function. Thus, we presented an efficient biological technique to control* Oscillatoria* in prawn ponds.
